# Biosimilars for anti-VEGF treatment of macular diseases: country and region reports

**Published:** 2025-01-31

**Authors:** Miriam Cano, Mangala Dhanapala, Mengtian Kang, Mapa Prabhath Piyasena, Rajiv Raman, David Yorston

**Affiliations:** 1 Head of Ophthalmology: Catholic University of Asunción School of Medicine, Asunción, Paraguay.; 2 Consultant Vitreo-Retinal Surgeon: Retina Research Unit, National Eye, Colombo, Sri Lanka.; 3 Ophthalmologist: Tongren Hospital, Beijing, China.; 4 Deputy Director: Vision and Eye Research Institute, Faculty of Health, Medicine and Social Care, Anglia Ruskin University, UK.; 5 Professor: Medical Research Foundation, Sankara Nethralaya & Indian Institute of Technology, Madras, Chennai, India.; 6 Consultant Ophthalmologist, Tennent Institute of Ophthalmology, Gartnavel Hospital, Glasgow, Scotland.

**Biosimilars can improve patients’ access to anti-VEGF treatment for macular diseases, but only if they are approved for local use and are readily available.**

**Figure:**
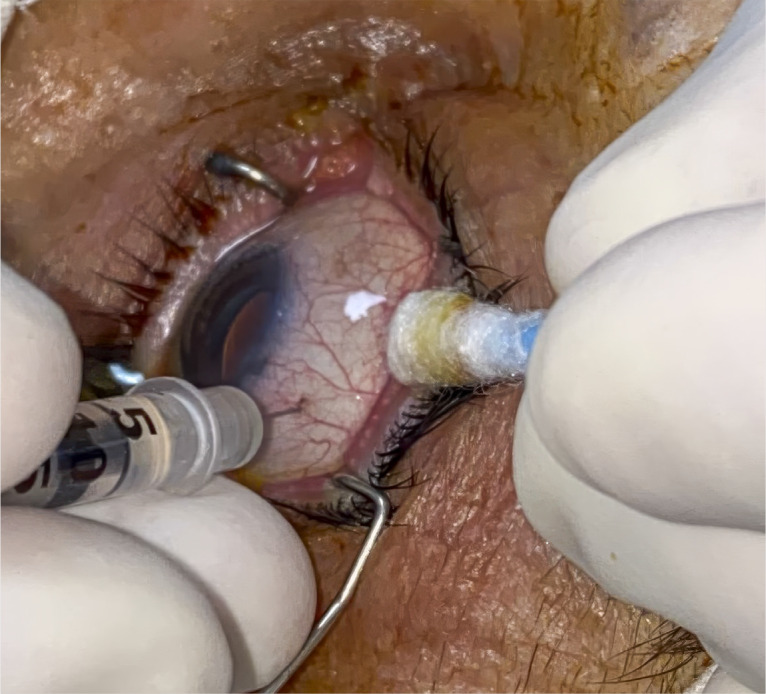
Anti-VEGF injection. PARAGUAY

© PABLO CIBILS CC BY-NC-SA 4.0

A major barrier to the treatment of macular diseases is the cost of anti-VEGF drugs. As the patents on these drugs expire, lower cost biosimilar drugs have become available. According to the World Health Organization, a biosimilar is a bio-therapeutic product which is similar in terms of quality, safety and efficacy to an already licensed (or reference) bio-therapeutic product.^1^ They are similar, not equal; their active ingredients are not identical to those of the reference product. The adoption of a biosimilar varies between countries due to factors such as regulatory approval, market acceptance, and health care policies.^1–2^

The following short reports from Latin America, Sri Lanka, and China give an insight into how the introduction of biosimilars has affected practice in these regions.

There are some common themes.

1. The biosimilars appear to be safe and effective, and no concerns were raised about the quality of locally produced biosimilar agents.
2. The price of biosimilars remains high, at 30–80% of the cost of the original product. This is a considerable saving, but it is still too much for many patients. We should remember that the cost of anti-VEGF treatment is not just the price of the drug; it includes lost wages every month, and travel to and from the injection centre.
3. Bevacizumab, a reference drug, remains the lowest cost treatment in many countries: by using one vial for multiple patients, the cost can be as low as US $10 per injection. However, a shared vial is not without risk, and large numbers of injections are needed to achieve this level of cost-effectiveness.

“The adoption of a biosimilar varies between countries due to factors such as regulatory approval, market acceptance, and health care policies.”

This is a rapidly developing field. At one time, intraocular lenses (IOLs) were too expensive for widespread use in low- and middle-income countries. However, locally manufactured IOLs are now cheaper than spectacles and their availability has transformed cataract surgery. We can hope that we will see similar developments with biosimilar anti-VEGF agents.

## China

An aging population, and the rising prevalence of diabetes, makes the need for anti-VEGF treatment of macular diseases particularly acute in China. Clinical guidelines recommend anti-VEGF drugs as the first-line treatment of age-related macular degeneration (AMD) and diabetic macular oedema (DMO). Lucentis (ranibizumab), Eylea (aflibercept), and Lumitin (conbercept) are the main anti-VEGF drugs in the Chinese market, and the price of one injection are Chinese yuan (CNY) 3,674 (US $502), CNY 4,150 (US $567), CNY 3,452 (US $470), respectively.

Zhuocuming (by Qilu Pharmaceutical Co), a biosimilar of aflibercept intraocular injection solution, was officially approved by the National Medical Products Administration (NMPA) for the treatment of adult patients with AMD and DMO. Zhuocuming is priced at CNY 2,970 (US $406) per injection. The phase 3 clinical trials are underway.

## Latin America

In Latin America, patients have access to all the anti-VEGF drugs on the market. However, many fail to complete their course of treatment due to the high cost.

Latin American countries Argentina, Brazil and Mexico are active members of the Pharmaceutical Inspection Co-operation Scheme (picscheme.org), which makes it possible for them to use biosimilars.^2^ Argentina and Brazil are the countries with the most biosimilars approved (more than 10 each). However, biosimilars are not often used in the region, because a single injection of a biosimilar could be over US $1,000 just for the medication,^3^ compared to the cost of US $50–70 for an intravitreal injection of bevacizumab.

As long as bevacizumab is cheaper than any biosimilar, there is no reason to use the latter. There is also currently more trust in bevacizumab thanks to the 2011 CATT Study,^4^ funded by the National Eye Institute in the USA, and the study published by the Pan-American Collaborative Retina Study Group in 2016.^5^

## Sri Lanka

Most of the anti-VEGF drugs used worldwide are available in Sri Lanka. The commonly used ones are bevacizumab (Avastin), ranibizumab (Lucentis), aflibercept (Eyelea) and faricimab (Vabysmo). Patizra, the ranibizumab preparation provided for low-and-middle-income countries by Novartis Indonesia, is also available in Sri Lanka.

Biosimilar bevacizumab preparations available in Sri Lanka are Avegra (by BIOCAD) and Abermy (by Biocon Ltd). The biosimilar for aflibercept that is locally available is Zaltrap (by Regeneron).

In clinical practice, one vial of bevacizumab is shared among 30 to 40 patients, hence the cost for an injection in the public sector is around Sri Lankan Rupee (LKR) 4,000 (US $13) per patient; this is given to patients free of charge. In the private sector, the cost per bevacizumab injection ranges from LKR 16,000 to 35,000 (US $54–118); the cost of Patizra injections ranges from LKR 90,000 to LKR 125,000 (US $303–420), and the cost per Vabysmo injection ranges from LKR 300,000 to LKR 325,000 (US $1,010–1,094).

Biosimilar anti-VEGF agents in Sri Lanka are little cheaper than the original product, but there are few designated local suppliers, which explains why these biosimilars are not more popular amongst retina specialists.

*See article on p24 for more on biosimilars in Sri Lanka.*

## India

India approved the world's first ranibizumab biosimilar, Razumab (by Intas Pharmaceuticals), in 2015. This biosimilar is approved for all conditions in which ranibizumab might be used, including neovascular (wet) AMD, DMO, retinal vein occlusions, and retinopathy of prematurity. Other notable biosimilars available in India include Ranibizumab-nuna (Byooviz), by Samsung Bioepis and Biogen, and several other candidates such as Ranizurel (by Reliance Life Sciences) and Ranieyes (by Lupin Limited), each having proven their efficacy and safety in clinical trials comparable to the reference drugs.^6,7^

Biosimilars are significantly cheaper than their branded counterparts, typically costing 35–50% less. This price reduction is due to the lower research and development costs associated with biosimilars compared to originator biologics.

Initial concerns about intraocular inflammation linked to early batches of Razumab were addressed by adjusting manufacturing processes, setting a precedent for robust quality controls in biosimilar production.^8^

Other drugs are in development. Several aflibercept biosimilars are poised to enter the market once patent protections expire. India's ability to develop and manufacture low cost biosimilars is expected to improve global access to treatments for retinal diseases.^9,10^
